# Characterizing cutaneous leishmaniasis in a conflict-affected region: a study from North Waziristan, Pakistan

**DOI:** 10.55730/1300-0144.5746

**Published:** 2023-10-10

**Authors:** Zahid ULLAH, Fazeelat SAMAD, Rahila BANO, Sarah ARIF, Sher ZAMIR, Nighat AZIZ, Hassan KHAN

**Affiliations:** 1 Department of Pathology, Gomal Medical College, Dera Ismail Khan, Pakistan; 2 Department of Physiology, Gomal Medical College, Dera Ismail Khan, Pakistan; 3 Department of Pharmacology, Gomal Medical College, Dera Ismail Khan, Pakistan; 4 Faculty of Pharmacy, Gomal University, Dera Ismail Khan, Pakistan

**Keywords:** *Leishmania tropica*, cutaneous leishmaniasis, North Waziristan, Pakistan

## Abstract

**Background/aim:**

Cutaneous leishmaniasis is an emerging tropical disease that remains a serious public health issue in Pakistan, particularly in North Waziristan. The current research was carried out to investigate the presence of cutaneous leishmaniasis in this region.

**Materials and methods:**

This prospective observational study was conducted from October 2018 to December 2020 at District Head Quarter Hospital Miranshah in North Waziristan with the collaboration of the Pathology Department of Gomal Medical College Dera Ismail Khan, Khyber Pakhtunkhwa. Needle aspirates were used for the microscopic Giemsa-stained slides. SPSS was used for data analysis.

**Results:**

Of the 5406 clinically-suspected cases, 2603(48.2%) were positive by microscopic examination. Of these 2603 patients, 1474 (57%) were male and 1129 (43%) were female. Most of the lesions were on the face, followed by upper and lower limbs. The 5–10-year age group had the highest percentage of 1167 (45%). A single lesion affected 96.6% of the patients, while 2.7% had double lesions and 0.7% had triple lesions. A high number of cutaneous leishmaniasis were seen from April to August, while the lowest number was seen November to December.

**Conclusion:**

This study provides extensive information in relation to the existence of cutaneous leishmaniasis in the North Waziristan district of Pakistan, as well as the detailed demographic features of those affected by the disease.

## 1. Introduction

Protozoan parasites of the genus *Leishmania* are responsible for the disease known as leishmaniasis (family Trypanosomatidae). These parasites can be spread by the bite of a female sand fly, especially those of the genera *Phlebotomus* and *Lutzomyia* [1]. There are 3 main types of leishmaniasis, comprising cutaneous, mucocutaneous, and visceral, which give rise to systemic disease. Visceral leishmaniasis is considered the most fatal in Africa. Leishmaniasis is an endemic disease that affects more than 12 million individuals worldwide in at least 88 different tropical and subtropical regions [2]. The most frequent type of leishmaniasis is cutaneous leishmaniasis, which produces lesions on the skin (most often ulcers) on exposed body areas and may result in permanent scarring, severe disability, or social stigma [3].

Around 95% of cutaneous leishmaniasis cases happen in the Americas, the Mediterranean Basin, the Middle East, and central Asia. In 2020, ten nations, comprising Afghanistan, Algeria, Brazil, Colombia, Iraq, Libya, Pakistan, Peru, the Syrian Arab Republic, and Tunisia, accounted for more than 85% of all new cases of cutaneous leishmaniasis. An estimated 600,000 to 1 million new cases globally are reported each year [4]. Cutaneous leishmaniasis is a rising public health concern in Asia, particularly in Pakistan. It was originally discovered in Pakistan in 1960 and was first only found in the northern hilly area of the country, but it has expanded and is now almost nationwide [5]. According to estimates, 400,000 cases of cutaneous leishmaniasis were reported in Pakistan in 2016, making up around 10% of all cutaneous leishmaniasis cases globally [6].

Cutaneous leishmaniasis is often documented from urban areas in Punjab, Sindh, Baluchistan, Azad Jammu Kashmir (AJK), Khyber Pakhtunkhwa (KP), and the adjacent tribal region, also known as the erstwhile Federally Administrated Tribal Areas (Ex-FATA) [7–9].

North Waziristan shares a northern border with Afghanistan, where cutaneous leishmaniasis is prevalent. Of the population in Afghanistan, 36% are at risk of contracting cutaneous leishmaniasis, making it one of the high burden nations for the disease [10].

Mass displacement of the population in and outside of the endemic area due to conflict, the lack of or insufficient access to public health facilities, poor living conditions, animal reservoirs, and climate and environmental changes are the factors that have added to the occurrence of cutaneous leishmaniasis. Therefore, this study was conducted to determine the distribution of cutaneous leishmaniasis in war-affected North Waziristan.

## 2. Material and methods

### 2.1. Study area and duration

This prospective observational study was conducted at a referral center for leishmaniasis of District Head Quarter (DHQ) Hospital Miranshah in North Waziristan with the collaboration of the Pathology Department of Gomal Medical College Dera Ismail Khan, Khyber Pakhtunkhwa (KPK), between October 2018 and December 2020.

**Figure 1:**
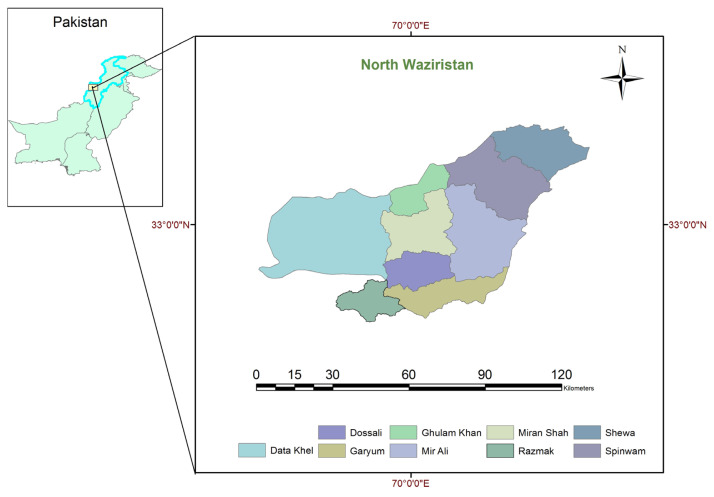
Map of the study area showing the 9 tehsils of District North Waziristan, Pakistan, using ArcGIS software.

District North Waziristan is the second largest in Ex-FATA, with an area of 4707 square kilometers and a population of 543,254. Administratively, North Waziristan is divided into 9 tehsils: Miranshah, Mir Ali, Razmak, Dossali, Gharyum, Data Khel, Ghulam Khan, Shewa, and Speenwam (Figure 1).

North Waziristan is bordered geographically in the north by Afghanistan, District Kurram, and District Hangu; in the east by tribal territories neighboring the Bannu and Karak Districts; in the south by the South Waziristan district; and in the west by Afghanistan as well.

### 2.2. Patient and smear examination

A total 5406 patients with clinically-suspected cutaneous leishmaniasis visited the District Head Quarter Hospital Lab of North Waziristan district area of Khyber Pakhtunkhwa. After obtaining both verbal and written informed consent in Pashto, Urdu, and English from the selected patients, samples were taken from them and they were photographed.

The lesion samples from the cutaneous leishmaniasis patients were taken using the needle aspiration method. The smear slides were fixed with alcohol and stained with Giemsa stain for parasitic examination. The stained slides were primarily examined using the oil immersion objective for *leishmania* parasite, followed by examination for the presence of the amastigotes of cutaneous leishmaniasis. The slides were secured in a slide box and reviewed by a microscopy professional. Patients with antileishmanial therapy in the proceeding weeks and those with other skin-related infections were excluded from the study.

### 2.3. Data analysis

Epidemiologic and statistical calculator OpenEpi, a web-based tool, was used to determine the sample size [11]. The Statistical Package for the Social Science (SPSS) was used for the statistical analysis.

## 3. Results

The lesion samples of 5406 patients with clinically-suspected cutaneous leishmaniasis were examined using the Geimsa staining technique. Of the 5406 patients, 2603 (48.2%) were positive by microscopic examination. Sociodemographic characteristics of the cutaneous leishmaniasis-positive patients are shown in Table 1. Of the 2603 patients, 1474 (57%) were male and 1129 (43%) were female. These patients were further divided into 7 age groups. It was seen that 1167 (45%) patients were in the 5–10-year age range, while 685 (26%) were under the age of 5 (Table 1).

In regard to education, the majority were illiterate 996 (52%). Moreover, 2289 (88%) patients were from the rural areas. The majority of the patients, 1614 (62%), had houses made of mud. Meanwhile, a large number of patients, about 1900 (73%), lived in houses with holes in them. In terms of profession, 170 (32%) patients were farmers, 149 (28%) were unemployed, and 133 (25%) were laborers (Table 1). Additionally, the frequency of cutaneous leishmaniasis was examined among the several areas/subdivisions of North Waziristan. The highest prevalence was found in Miranshah at 41.1%, followed by Ghulam Khan at 28.8%, and MirAli at 13.6% (Table 2).

**Figure 2:**
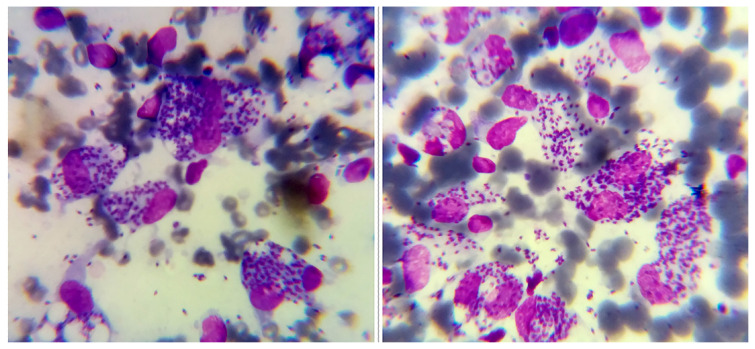
Microscopic Giemsa-stained slides (1000X) showing both the intracellular and extracellular amastigotes of cutaneous leishmaniasis patients of North Waziristan.

During examination of the Giemsa-stained smears of the skin scrapings and aspirates, the amastigotes were seen as round or oval structures containing nuclei and kinetoplasts (Figure 2).

**Figure 3:**
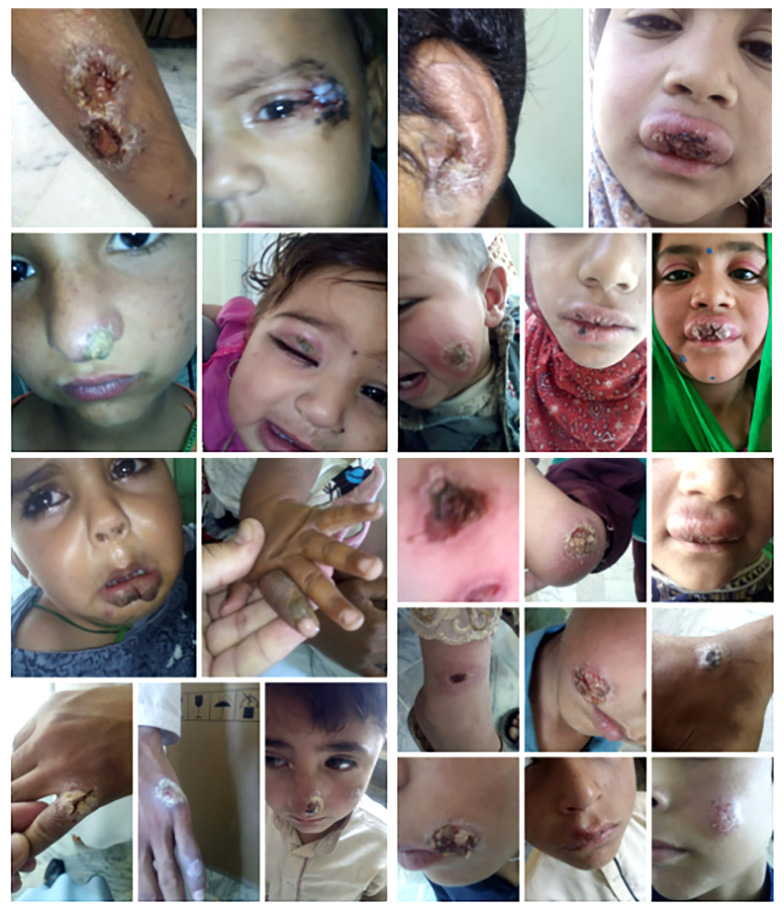
Cutaneous leshmeniasis lesions: on hands, arms, and face from the area.

The majority of the cutaneous leishmaniasis lesions were located on the uncovered body parts, such as the face and upper and lower limbs (Table 3 and Figure 3).

## 4. Discussion

Cutaneous leishmaniasis is a growing tropical disease that continues to constitute a serious public health risk in Pakistan, particularly in the KPK and tribal regions. Estimating the prevalence of cutaneous leishmaniasis infection in Pakistan has been difficult due to the wide range of data available on the incidence and distribution of the disease, and the number of cases confirmed by microscopy, making it impossible to determine whether the endemicity of the disease is increasing, decreasing, or remaining stable [12,13].

The current study was conducted in North Waziristan, a high-altitude region on the border of Afghanistan, where cutaneous leishmaniasis is endemic. The study results showed 48.2% smear positivity of cutaneous leishmaniasis in the human population of North Waziristan. Similarly, a study conducted including local and Afghan refugees in the Lower Dir district of KPK revealed 51% positivity [14], while there was 50.6% positivity in Baluchistan [6]. Another study in North Waziristan reported 53.7% positivity for cutaneous leishmaniasis [15]. Over the last several decades, this region has been politically unstable due to ongoing military operations against terrorists. The instability in this area has had a devastating effect on the public health system there. Additionally, the high rate of cutaneous leishmaniasis in the region is likely attributable to its closeness to Afghanistan, an area where the disease is prevalent. According to the current research findings, males were affected by cutaneous leishmaniasis more than females. This finding is supported by the opinions of some national and international experts [15–20]. A higher proportion of males were diagnosed with cutaneous leishmaniasis than females, which may be due to a combination of factors, such as greater exposure to the disease because men tend to work outdoors and are less likely to wear protective clothing, leading to a higher incidence of infected sand fly bites, or less frequent medical attention because women may have less access to health-care facilities. Moreover, females are better at controlling cutaneous leishmaniasis infection owing to the activation of the Th1 immune response by estrogen, whereas androgens inhibit the immune response in men, making them more susceptible to disease [21]. According to the present research, 45% of the cutaneous leishmaniasis infections were in children aged 5–10 years, followed by 26% in children aged 5 years, which might be linked to the children having compromised immune systems and food instability. Other studies have also supported this pattern [6,20,22,23]. Regarding education levels, the majority of the patients in the present study were illiterate, because education and awareness play a crucial role in the control of cutaneous leishmaniasis in individuals and mothers, which is in agreement with the studies of Hejazi et al. [24] and Irum et al. [25]. The preponderance of the cutaneous leishmaniasis patients were male farmers in terms of their profession in the present study. In these cultures, the ideal male is strong, daring, a risk-taker, and able to endure physical sacrifices and danger. Moreover, males who work as framers participate in outdoor pursuits like fishing and hunting, which are observations also supported by other studies [26–28]. According to the findings herein, only 38% of the cutaneous leishmaniasis patients lived in cement houses, whereas 62% lived in non-cemented houses with roofs composed of mud mixed with straw and wood. Patients living in houses made of mud were more impacted than those living in cement houses. Cracks and holes in mud walls may harbor sandflies in the research region. Previously, mud-plastered walls were linked to the sandfly vector density [29,30]. The present research found that in North Waziristan, rural regions reported a higher prevalence of cutaneous leishmaniasis cases than urban areas. This is in line with several previous reports [21,31]. In the present study, most of the cutaneous leishmaniasis patients had a single lesion (96.5%) and remaining had 2 to 3 lesions. Previous studies support these findings [32–34]. In the current study, the face was the part of the body most often affected with cutaneous leishmaniasis lesions, followed by the arms and legs, because these are the most exposed parts of the body and are easily accessible to sandfly bites. Other researchers reported similar findings [6,35–37^1^]. The seasonal distribution of cutaneous leishmaniasis cases, measured over 2 years and 3 months in the current study, markedly increased from April to August, while the lowest rates of disease distribution were seen from November to December. Diverse studies have identified varying transmission patterns that point to seasonal tendencies in various geographic settings [38–41].

The high prevalence of cutaneous leishmaniasis in North Waziristan may be attributed to several possible causes. These include inadequate vector-control measures, inadequate healthcare infrastructure, a lack of education, socioeconomic characteristics such as poverty, internal mobility due to seasonal changes or military operations, and restricted access to healthcare services.

## 5. Conclusion

The prevalence of cutaneous leishmaniasis is higher in the study region than in other areas. The high incidence of cutaneous leishmaniasis poses a risk to public health and challenges public health professionals. The cutaneous leishmaniasis burden in this region can certainly be reduced with the help of improved political stability, healthcare infrastructure, vector-control measures, rapid and accurate parasite detection methods, species-specific treatment for cutaneous leishmaniasis, a public awareness campaign against, and an essential prevention approach for cutaneous leishmaniasis.

## 6. Limitations

This study had 2 limitations. First, the study was significantly constrained by the lack of resources and funding. Therefore, it is highly recommended for future research to employ more advanced molecular-based assays for species typing in this area. Second, due to security concerns, the researchers faced limited access to the study district, which hindered in-depth investigations into the function of sandfly vectors.

## Figures and Tables

**Table 1:** Demographic characteristics of cutaneous leishmaniasis (n = 2603).

Factors		N (%)
Sex	Male	1474 (57%)
	Female	1129 (43%)
Age, years	<5	685 (26%)
	5–10	1167 (45%)
	11–15	220 (8%)
	16–20	126 (5%)
	21–25	71 (3%)
	26–30	103 (4%)
	30+	231 (9%)
Level of education (n = 1918)	High school	633 (33%)
	College	211 (11%)
	University	78 (4%)
	Illiterate	996 (52%)
Locality	Local	2579 (99%)
	Nonlocal	24 (01%)
Profession (n = 532)	Farmer	170 (32%)
	Labor	133 (25%)
	Government servant	80 (15%)
	Jobless	149 (28%)
House type	Cement	989 (38%)
	Mud	1614 (62%)
Presence of holes in the house	Yes	1900 (73%)
	No	703 (27%)
Habitat	Urban	314 (12%)
	Rural	2289 (88%)

**Table 2:** Subdivisions/areas with cutaneous leishmaniasis (n = 2603).

Area	N = (%)
Miranshah	1069 (41.07%)
Ghulam Khan	750 (28.81%)
Mir Ali	553 (13.56%)
Dossali	130 (4.99%)
Garyum	19 (0.73%)
Data Khel	52 (2.00%)
Razmak	24 (0.92%)
Shewa	4 (0.15%)
Speenwam	178 (6.84%)
Outside of North Waziristan	24 (0.92%)

**Table 3:** Number of lesions and site-wise distribution of cutaneous leishmaniasis (n = 2603).

Lesion	Site	N (%)
Single	Head	1 (0.04%)
	Face	1601 (61.58%)
	Ears	21 (0.81%)
	Neck	3 (0.12%)
	Trunk	2 (0.08%)
	Hands	538 (20.67%)
	Legs	346 (13.29%)
Double	Shoulders	2 (0.08%)
	Face, hands	29 (1.11%)
	Face, legs	19 (0.73%)
	Hands, legs	25 (0.96%)
Triple	Ears, hands, legs	1 (0.04%)
	Face, hands, legs	13 (0.50%)
